# It's time to address fear of cancer recurrence in family caregivers: usability study of an virtual version of the Family Caregiver—Fear Of Recurrence Therapy (FC-FORT)

**DOI:** 10.3389/fdgth.2023.1129536

**Published:** 2023-08-21

**Authors:** Jani Lamarche, Angélica Cusson, Rinat Nissim, Jonathan Avery, Jiahui Wong, Christine Maheu, Sylvie D. Lambert, Andrea M. Laizner, Jennifer Jones, Mary Jane Esplen, Sophie Lebel

**Affiliations:** ^1^ Faculty of Social Sciences, School of Psychology, University of Ottawa, Ottawa, ON, Canada; ^2^ Department of Supportive Care, Princess Margaret Cancer Centre, University Health Network, Toronto, ON, Canada; ^3^ Department of Psychiatry, Temerty Faculty of Medicine, University of Toronto, Toronto, ON, Canada; ^4^ Cancer Chat De Souza Institute, University Health Network, Toronto, ON, Canada; ^5^ Ingram School of Nursing, McGill University, Montreal, QC, Canada; ^6^ St. Mary's Research Centre, St. Mary's Hospital Center, Montreal, QC, Canada; ^7^ Research Institute of the McGill University Health Centre, McGill University Health Centre, Montreal, QC, Canada

**Keywords:** usability, fear of recurrence, cancer, intervention, e-health, family caregivers

## Abstract

**Background:**

Family caregivers of cancer survivors experience equal or greater levels of fear of cancer recurrence (FCR) than survivors themselves. Some interventions have demonstrated their ability to reduce FCR among cancer survivors and dyads (patient and caregivers). However, to date, no validated intervention exists to focus solely on family caregiver's FCR.

**Objectives:**

This study aimed to (1) adapt the evidence-based in-person Fear Of Recurrence Therapy (FORT) for family caregivers (referred here in as FC-FORT) and to a virtual delivery format and (2) test its usability when offered virtually.

**Methods:**

The adaptation of FC-FORT was overseen by an advisory board and guided by the Information Systems Research Framework. Following this adaptation, female family caregivers and therapists were recruited for the usability study. Participants took part in 7 weekly virtual group therapy sessions, a semi-structured exit interview and completed session feedback questionnaires. Therapists were offered a virtual training and weekly supervision. Fidelity of treatment administration was assessed each session. Quantitative data were analyzed using descriptive statistics. Exit interviews were transcribed verbatim using NVivo Transcription and coded using conventional content analysis. Results were presented back to the advisory board to further refine FC-FORT.

**Results:**

The advisory board (*n* = 16) met virtually on 7 occasions to adapt FC-FORT (i.e., patient manuals, virtual format) and discuss recruitment strategies. Minor (e.g., revised text, adapted materials to virtual format) and major adaptations (e.g., added and rearranged sessions) were made to FC-FORT and subsequently approved by the advisory board. Four family caregivers and three therapists took part in the first round of the usability testing. Six family caregivers and the same three therapists took part in the second round. Overall, participants were very satisfied with FC-FORT's usability. Qualitative analysis identified 4 key themes: usability of FC-FORT, satisfaction and engagement with content, group cohesion, and impact of FC-FORT. All participants indicated that they would recommend FC-FORT to others as is.

**Conclusions:**

Using a multidisciplinary advisory board, our team successfully adapted FC-FORT and tested its usability using videoconferencing. Results from this study indicate that the efficacy and acceptability of FC-FORT are now ready to be tested in a larger pilot study.

## 1. Introduction

Fear of cancer recurrence (FCR) is defined as the fear, worry, or concern that cancer may come back or progress (1). FCR is common and manifests itself on a continuum with an estimated 59% of survivors reporting moderate to severe levels of FCR (2) (i.e., clinical FCR). Clinical FCR is associated with impairment in functioning, psychological distress, sleep difficulties, stress response symptoms, and lower QOL (3–9). Common risk factors for FCR include younger age, gender (with women being more prone than men) (2), and presence of somatic symptoms (such as pain and fatigue) (10). Furthermore, FCR seems to be common and persistent across various types and stages of cancer (2). To date, there is evidence from two meta-analyses (11, 12) that clinical FCR can be reduced among cancer survivors by either group or individual therapy with small to moderate effect sizes [Hedges's g ranging from 0.28 – 0.33 (12) and pooled effect size of *g* = −0.36 11] and evidence of sustained improvements at follow-up (on average 8 months post-therapy). While evidence for the efficacy of interventions designed to address FCR in cancer survivors has been demonstrated, to date, there have been few attempts to offer these treatments to family caregivers (FC) despite FC experiencing similar levels of FCR than survivors.

FC are defined as family members who provide unpaid support and play an integral role in the treatment and care of cancer patients & survivors (13–15). Approximately 50% of FC experience levels of FCR equal or greater than those reported by cancer survivors (16). Similarly to survivors, FCR in FC is persistent, associated with lower QOL, lower functioning, and higher psychological distress (9, 17). A recent qualitative exploration (18) of FCR in FC identified three key themes including fear that the patient will suffer, protecting the patient from a potential recurrence and/or cancer-related distress, and a sense of unpreparedness or uncertainty (in relation to losing the patient). Results also demonstrated that FC felt an important sense of personal responsibility for the life of the patient and that this was a driving factor for both FC and patient FCR. Furthermore, studies suggest that, in couples, levels of FCR experienced by one partner influences FCR levels experienced by the other partner (9, 19–21). Thus, it appears that treating FCR in FC could impact FCR in both FC and survivors.

Many interventions have been developed to address the needs of FC of cancer survivors (22–24). Generally, these include psychoeducation, skills training and counselling in individual, group, and paired settings. Results from two meta-analyses (22, 23) suggest that interventions dedicated to FC generally have small to medium effect sizes in reducing FC burden, alleviating psychological symptoms, and improving FC coping capabilities, self-efficacy and QOL overall. In addition, to date, three dyadic (for survivors and FC) interventions (16) have been developed to address FCR in FC. Whereas results from one study (25) suggested a significant reduction of FCR in survivors' post-intervention (compared to the control group), no significant decline was demonstrated in FC's FCR. Thus, this is the first attempt to develop and evaluate an intervention to individually address FCR in FC. In considering the adaptation of the proposed pilot project, it is important to account for the consistent evidence that FC often cannot access in-person services due to a variety of constraints. Previous in-person therapy studies for FC have reported difficulty to reach FC and had low attendance and high attrition rates (26). Furthermore, traditional in-person interventions may also not be feasible in the current COVID-19 era (27). Therefore, e-health interventions could provide a more viable option for this specific population.

Multiple studies have suggested that therapist guided e-health interventions could be as efficient as traditional in-person therapy and that participants perceive the same levels of satisfaction and therapeutic alliance (28–34). Virtual support groups via videoconferencing are suggested to be comparable to in-person interventions as it enables real-time interactive face-to-face exchange, while drawing participants that may otherwise not be able to access support (35). Indeed, a systematic review (36) found e-Health interventions to be feasible, usable, and acceptable for FC of cancer survivors. Another systematic review (37) identified that, compared to care as usual, therapist led e-Health interventions effectively reduced symptoms of depression and improved quality of life of FC of cancer survivors. Given the many constraints experienced by FC (13), including accessibility and financial, e-Health interventions represent a great alternative and, perhaps, a preferable option to in-person therapies.

Given the limited evidence-based interventions developed for FC to manage their FCR, our team set out to adapt and pilot an intervention to better inform a larger randomized control trial. The aim of the present study was to adapt the Fear Of Recurrence Therapy (FORT) (38–40) to FC and to a virtual format and test its usability. FORT is a standardized and manualized therapist led in person intervention. It consists of 6 consecutive weekly group or individual sessions of 90–120 min each and weekly assigned homework (38, 39) (See Table 1 for a detailed description of each session). Sessions 1 to 4 aim to build skills and coping strategies in preparation for session 5 where participants are asked to identify and expose themselves to their worst fears related to FCR. Session 6 serves a wrap up and last chance to ask clarifying questions. The key goals of FORT include helping women: (1) distinguish worrisome symptoms from benign ones; (2) identify FCR triggers and inappropriate coping strategies; (3) facilitate the learning and use of new coping strategies, such as relaxation techniques and cognitive restructuring; (4) increase tolerance for uncertainty; (5) promote emotional expression of specific fears that underlie FCR; and (6) re-examine life priorities and set realistic goals for the future. FORT is based on a blended theoretical model of FCR (41) that aims to address key vulnerability factors such as internal and external triggers, exaggerated perceived risk of recurrence, hyper-focus on ambiguous physical sensations, maladaptive coping, uncertainty around cancer and its treatments or care, intolerance of uncertainty, and beliefs about the benefits of worrying about one's health. Key components include: (1) principles of group therapy (e.g., promoting group cohesion by facilitating participants' self-disclosure of their FCR), (2) Cognitive Behavioural Therapy-based techniques (e.g., cognitive restructuring), and (3) elements of existential therapy (e.g., identifying and addressing fears related to death and dying). FORT (38, 40) has previously demonstrated its effectiveness at reducing FCR (moderate effect size; *d* = −0.53) and secondary outcomes [triggers, (*d* = −0.415), coping (*d* = −0.244), cognitive avoidance (*d* = −0.424), QOL mental health (*d* = 0.165)] in women cancer survivors (40) with sustained improvements at a 3-month follow up. Additionally, a series of case studies of cancer survivors receiving the individual FORT intervention via videoconference (42) suggested acceptability and usability. Given that FORT has so far only demonstrated its effectiveness with female cancer patients/survivors and that nothing is known about its potential impact on FCR in men or non-binary individuals, the present study focuses specifically on women FC. Furthermore, research consistently indicates that women carry a heavier caregiver burden than men (43).

**Table 1:** Overview of FORT sessions.

Session 1: Introduction to the group, learning new skills to deal with FCR (60–90 min)	• Introduction by each participant with a focus on their experience with FCR. • Introduce ABC model of cognitive behavioural therapy. • Introduce FCR model. • Introduce notion of cognitive restructuring and identify triggers. • Teach progressive muscular relaxation. • **Homework:** Practice progressive muscular relaxation daily; complete thought record and challenge maladaptive thinking about fear of cancer recurrence.
Session 2: Providing information, increasing tolerance for uncertainty (60–90 min)	• Prepare questions for visit from health care professional who will be providing education about signs of recurrence in session 3. • Help participants deal with the fact that uncertainty can never be completely eliminated. • Discuss ways of regaining a sense of control. • Teach the use of calming self-talk. Provide participants with relaxation files and instruct them to use calming self-talk phrases when appropriate. • **Homework**: Listen to relaxation files every day; practice calming self-talk; complete thought record and challenge maladaptive thinking about fear of cancer recurrence and prepare questions for the nurse visit.
Session 3: Building your coping skills (60–90 min)	• Visit from health care professional. • Increase tolerance for uncertainty by discussing acceptable level of worry. • Challenge faulty beliefs about benefits of worry. • Decrease maladaptive coping strategies. • Teach guided imagery. • **Homework**: Practice guided imagery daily; continue challenging faulty beliefs about benefits of worry; complete thought record adding a column for behaviors to monitor the kinds of coping strategies participants are adopting.
Session 4: Getting deeper into underlying fears (60–90 min)	• Provide psychoeducation about worry and the need for exposure to worse fears. • Promote emotion expression and confront specific fears that underlie FOR. • Write down worse fear scenario. • Teach mindfulness exercise. • **Homework:** Read worst case scenario every day and then do a self-care activity. Practice mindfulness exercise daily.
Session 5: Moving beyond specific fears (60–90 min)	• Review exposure to worst case scenario exercise. • Discuss ways of coping with some of the feared outcomes. • Promote expression of feelings of demoralization. • Encourage participants to become re-engaged with important life goals, people or activities they may have given up. • Discuss what meaning the future and planning now have for them. • Teach mindfulness exercise. • **Homework:** Write down goals and priorities for the future.
Session 6: Review and conclusion (60–90 min)	• Review all content covered. • Discuss future goals. • Setting new priorities. • Promote the expression of saying good-bye to the group and provide closure.

## 2. Methods

### 2.1. Adaptation framework

The adaptation of the Family Caregiver—FORT (FC-FORT) was guided by the *Information Systems Research Framework* (44)*.* This framework consists of three components that simultaneously influence each other: the Rigor Cycle, the Design Cycle, and the Relevance Cycle.

In the Rigor Cycle, the advisory board used its expertise and the existing FC literature to define and inform the content needing adaptation to FC concepts (e.g.: protective buffering, self-care, communication with cancer patients/survivors) and to a virtual format. Findings from the Rigor Cycle then informed the Design Cycle where the adapted FC-FORT program was developed (adaptation of the patient and therapist manuals, modifications to deliver the intervention virtually). This first version of FC-FORT was then put into the Relevance Cycle in the real-world environment where it was field tested by FC and therapists (See below Round 1). The results gathered in the first round were presented back to the advisory board who assessed that a second Design Cycle was needed. Further adaptations (see Table 2) were then made to FC-FORT and the second version of FC-FORT was put into the Relevance Cycle for a second round of usability testing (See below Round 2). The results gathered were then presented back to the advisory board who deemed to have a satisfactory version of FC-FORT. In a future part of this project, FC-FORT will be further evaluated in a pilot study and results will become incorporated in the Rigor Cycle as an addition to the knowledge base.

**Table 2:** Suggestions and adaptations made to FC-FORT.

Categories of proposed changes	Advisory board	Family caregivers/therapists (Round 1)
Content	Separate the amounts of psychoeducation content and theories from Session 1 into two sessions Reduce jargon, soften language, and make models more accessible Add quotes, images, graphs, colours to help with understanding and differentiation Add Youtube videos to review during the group Add therapist dialogue on myths about worrying and helpful vs. unhelp worries Add example of “fear of dogs” to demonstrate avoidance and exposure frameworks Add example scenario to Session 5 to guide participants Reduce expectations for homework and attendance (compard to FORT) Modify mindful eating exercise (ask participants to bring a small bite size snack). Send email reminder about bringing the food. Send a reminder prior to Session 5 to prepare participants to the difficulty of the session	Soften language and reduce expectations (especially homework) Review and reorganize the content of sessions 2, 3, 4 Remove health care provider's visit
Additional sections	Add a section on communication with loved one with cancer to address protective buffering Add a section on self-care to address FC needs (i.e., sleep, happiness) and/or difficulties taking time for themselves (i.e., guilt) Add a section about FC objective knowledge on FCR within their family member's cancer experience, identifying knowledge gaps, difficulties in obtaining additional information, problem solving how to properly obtain the information, naviguating and/or utilizing the patient's health care team, how to ask questions or discuss with health care providers • Adding an FAQ in the workbook Add a 7th session to accommodate the content Addition of a follow up check-in (1 month later) or a booster session^a^	
Additional information	Overall, increase examples and explanations so the workbook can stand alone and be referred to lateron Given the diversity of cancers within the group, make the healthcare provider's visit more general and focused on FC Ask the healthcare provider to leave their contact information in the event that FC have additional questions^a^	Increase examples and explanations so the workbook can stand alone and be referred to lateron
Technical features	Use a virtual group format Use max session length of 90 min Add a breakout room 30 min before the start of each session to create a space where FC can connect Add a breakout room to session 5 so therapists can connect with FC individually Biweekly sessions^a^ Record each session in order to send to participants who may have missed a session and would like to review^a^ Ask participants to send their weekly homework to therapists to increase completion^a^ Grouping caregivers by cancer types^a^	Remove the individual breakout room in Session 5 Establish more rigid group norms regarding the use of the chat function Ensure that participants use their camera every session Increase session length to 120 min
Accessories	While the intervention should use a virtual format, participants should receive a paper version of workbook that they can write in and keep Send audiofiles via email each week so participants can use them for their homework As much as possible, therapists should share their screens with participants	Group all audiofiles in one place (e.g.: google Drive) and send them at the beginning
Peer support	Participants should be encouraged to share the contact information at the end of the intervention	
Additional resources	Add resources at the back of the workbook	

^a^ This suggestion was taken into consideration but ultimately not applied to FC-FORT.

### 2.2. Adaptation of the Family Caregiver—Fear Of Recurrence Therapy (FC-FORT)

A multidisciplinary advisory board (16 members) comprised of 8 health researchers with expertise in psychosocial oncology and caregiving, 2 clinical psychologists, two therapists with experience in virtual support group formats and psychosocial oncology, and 4 female FC (having taken or currently taking care of partners) was created. FC were recruited through The Ottawa Hospital's Patient Engagement Office, Cancer Chat Canada and Twitter. Interested FC met with the study's research coordinator and lead psychologist to discuss their eligibility, advisory board expectations and their involvement. The *Patient Engagement Framework* from the *Strategy for Patient-Oriented Research* (SPOR) developed by the Canadian Institutes of Health Research (45) was used to guide patient engagement. SPOR is guided by four principles: inclusiveness, support, mutual respect, and co-building. Core areas of engagement include: (1) Patient Engagement in Governance and Decision-Making, (2) Capacity Building for Patient Engagement, and (3) Tools and Resources. See Table 3 below for examples of how this study included these core areas.

**Table 3:** Inclusion of SPOR core areas.

Patient engagement in governance and decision-making	Capacity building for patient engagement	Tools and resources
• Detailed terms of references when onboarding • Clear roles and expectations within advisory board • Inclusion in decisive parts of the process (i.e., adaptation, review of results, recruitment strategies, approval of final intervention)	• Obtained funding for the project and offered stipends to FC • Flexible schedule (i.e., dates/times/videoconferencing) to ensure FC could be present • Authorships on presentations and papers	• Training and orientation for advisory board and review process • Provided education on FCR and FORT • Video capsules

**Figure 1:**
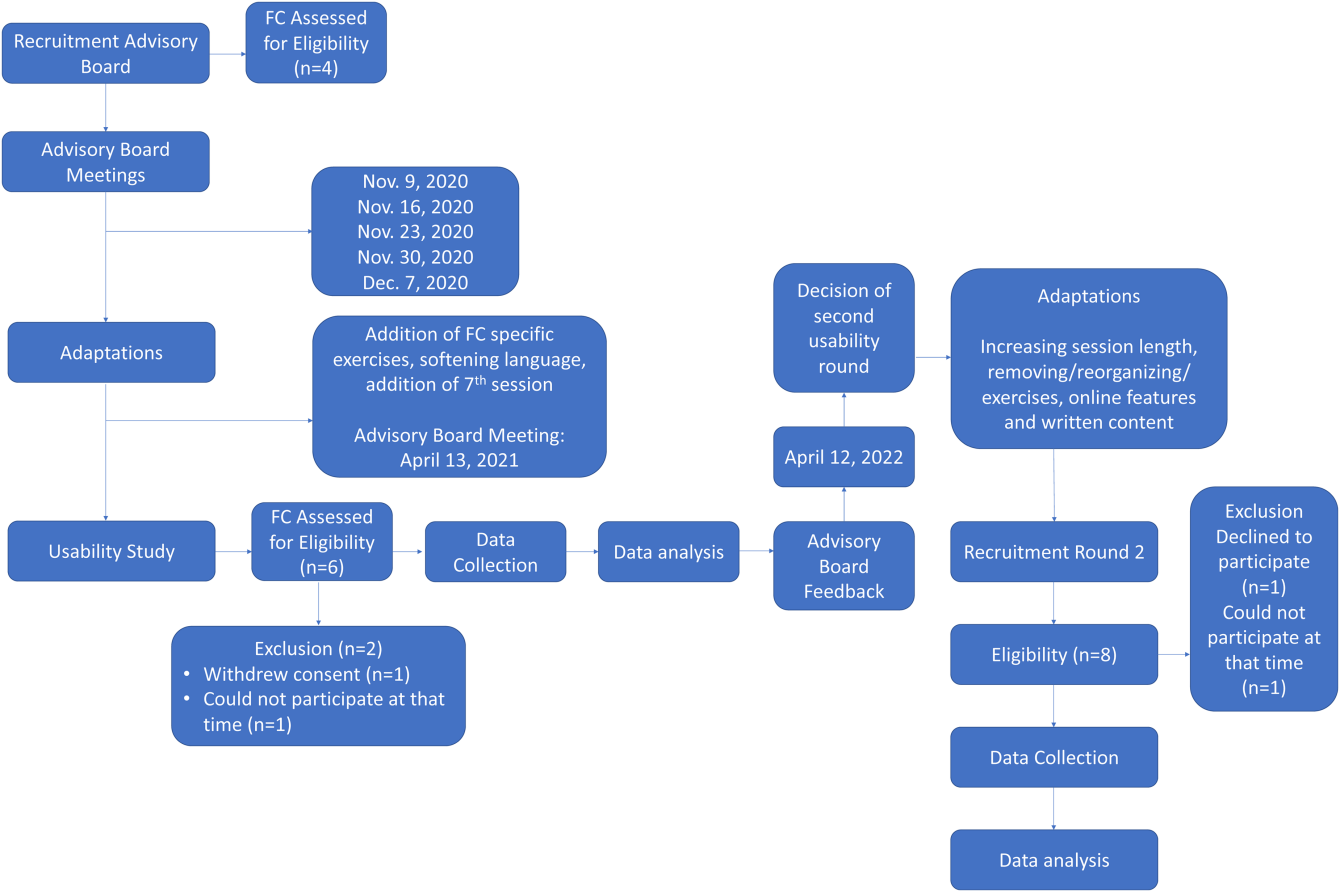
Flow diagram.

This advisory board met on Zoom on seven separate occasions, from November 2020 to April 2021, to oversee the adaptation of the FORT patient manual and provide feedback on recruitment strategies and the study's questionnaire package. The initial meeting focused on the study objectives, overall timeline, and roles and responsibilities. Following the initial meeting, members of the advisory board were asked to review two sessions each week to determine appropriateness of FCR content for caregivers and online format. Brief video capsules (10–15 min), created by the study's lead psychologist, explaining each of the FORT sessions were provided to facilitate their understanding and review. Feedback was received either by email or live during the meetings. Each meeting was recorded and reviewed by the research coordinator to confirm the feedback received. All aspects of sessions (i.e., exercises, text, images, graphics, examples) were reviewed with the entire advisory board and group discussions were held regarding their relevance to FC experiencing FCR, readability, understanding and acceptability. Discussions surrounding themes from the existing literature on FC's fears (i.e., fear of loved one suffering or dying, protectiveness, responsibility for patient's wellbeing), needs and unique challenges related to FCR were also conducted and considered when informing the adaptation to FC. The advisory board was consulted after each round of the usability study (described below) to discuss next steps (i.e., readiness for testing in a randomized controlled trial or need for an additional round of usability study). See Figure 1 for a Flow Diagram of the present study.

### 2.3. Adaptations of FORT to FC-FORT

The general structure, principles, key goals and components of FC-FORT remained very similar to FORT. Nonetheless, as expected, input from our advisory board led to some minor and major adaptations. Major adaptations included offering the intervention virtually (rather than in person), incorporating exercises aimed at addressing FC's self-care, overcoming protective buffering (i.e., the tendency to withhold sharing painful feelings to not burden others) (46, 47) by having difficult conversations with loved ones regarding FCR, and discussing (i.e., identifying knowledge gaps about family member's cancer experience, additional information needed and problem solving how to obtain it) and optimizing use of loved ones' health care teams (i.e., identifying lack of knowledge on FCR, identifying key members of the health care team, navigating meetings with health care providers while being a caregiver). Minor adaptations included softening the language of the patient workbook to represent caregivers' realities, additional examples and stand-alone explanations specifically related to FC fears and experiences, addition of YouTube videos, making the workbook more visually pleasing, and virtual breakout rooms before each session (to encourage FC to mingle amongst eachother) and in session 5 (allow group facilitators to check-in with participants). The inclusion of this supplementary content also led to the addition of a 7th session. Please see Table 2 for a list of all suggestions and adaptations.

### 2.4. Usability study

#### 2.4.1. Design

Participants took part in the FC-FORT intervention facilitated by two therapists (and 1 back up therapist). FC and therapists completed session feedback questionnaires, administered via Qualtrics, which collected data about specific aspects of each of the FC-FORT sessions. Post-intervention, semi-structured exit interviews, partially informed by the feedback questionnaires, were conducted with therapists and FC virtually using Zoom. These interviews provided a more general understanding of FC's experiences and general impact of FC-FORT, where difficulties arose, and how well FC-FORT met FC expectations and objectives. An initial round of the usability study was conducted and results were presented to the advisory board. Following this, adaptations were made to FC-FORT and a second round of usability testing was deemed necessary.

#### 2.4.2. Participants

Canadian adult female FC were eligible to participate if they: (1) cared for an adult survivor of any cancer type (stages I-III) who had completed their treatments and not experienced a recurrence, (2) had a score of 13 or greater on the Caregiver version of the Fear of Cancer Recurrence Inventory-ShortForm (48, 49), (3) spoke English, (4) had access to a computer and stable internet connection, (5) were not participating in another therapist-led psychosocial support group or a peer-led support group related to FCR, and (6) were not experiencing undermanaged mental health issues judged to be clinically contra-indicated and/or likely to affect the group work. Canadian therapists, recruited to administer FC-FORT, were deemed eligible if they: (1) were registered professionals in counselling or psychotherapy, (2) had at least 5 years of experience in psychosocial oncology, and (3) had led at least one support group. Participants were recruited through Cancer Chat (a virtual support group program of de Souza Institute in Canada), the Princess Margaret Cancer Centre in Toronto, Canadian cancer societies, social media, and community support partners across Canada. The same recruitment procedures were used for both rounds of the usability study.

#### 2.4.3. Procedure

The University of Ottawa's Office of Research Ethics and Integrity provided approval for this study (reference number H-05-20-5584). Interested FC contacted the research coordinator to be screened for eligibility, and, if appropriate, completed the consent form and prepared for the group work (i.e., reviewed expectations, assessed appropriateness of group) (38, 39). In addition, each FC was asked to provide further information regarding specific fears related to cancer recurrence and their caregiver experiences. Pre-intervention, FC received a standardized electronic or paper manual describing each session's activities and assignments, were asked to complete a sociodemographic questionnaire and provided their emergency contact information. Therapists were provided with a standardized FC-FORT facilitator's manual and received a 2h virtual training on FC-FORT by one of the study psychologists. FC and therapists then completed the 7-week FC-FORT intervention. Two days prior to each session, the research coordinator sent FC a reminder, the necessary materials if needed (e.g., audiofiles for the relaxation exercises) and the Zoom link via email. Participants were asked to inform the research coordinator via email if they needed to miss a session. On the days of the session, the research coordinator opened the Zoom meeting 30 min prior to the start of each session and was responsible for letting participants into separate break out rooms for FC (offer a chance to get to know eachother) and therapists (brief session prep or check-in). Therefore, outside of the 2 h sessions, FC and therapists had no direct contact with eachother. The research coordinator also managed the Zoom during the sessions (i.e., breakout rooms, recording the sessions, sharing permissions). During each session, participants were strongly encouraged to keep their cameras and microphones on, to use the chat feature for group messaging and private messaging between participants was discouraged (as it could not be monitored). Therapists began each session with a weekly check-in (i.e., how participants were doing), reviewed the weekly homework, and then moved through each of the workbook exercises. Participants were invited to jump in when pertinent, but therapists also made sure to engage participants that spoke less (i.e., round tables, asking FC directly). In addition, therapists separated the content of each session equally and shared their screens for relevant exercises (i.e., to show graphs or Youtube videos) or to ensure that participants were all at the same place in the workbook. While one therapist guided the content of the group, the other monitored the Zoom chat and participants wellbeing. Participants were offered a short break (5–10 min) midway through each sessions. Therapists finished each session with grounding and check-out (i.e., how participants were feeling) exercises. After each session, therapists and FC were asked to complete a feedback questionnaire (50) via Qualtrics. These questionnaires consisted of closed and open-ended questions aimed at assessing the usefulness (i.e., sessions provide information and tools that help understand and manage FCR), usability (i.e., user-friendly, easily understandable, can be followed-along with ease), desirability (i.e., visually appealing, organization of the information is clear, information is presented as a positive addition to the user experience), value (i.e., sessions provide a deeper understanding of FCR and valuable tools to manage FCR), accessibility (i.e., easy to follow along and navigate, information is relevant and easy to absorb), the virtual delivery format (i.e., breakout rooms) and features (i.e., exercises that were helpful and could be referred to at a later time), overall satisfaction, the general readiness of each session and potential additions, deletions or changes. The questionnaire is based on the user experience honeycomb developed by Morville & Sullenger in 2010 (51) and was adapted from Tan-MacNeill & colleagues' (52) usability study. Close-ended questions were rated on a 5-point Likert scale (“Strongly Agree” to “Strongly Disagree”; “Extremely Ready” to “Not At All Ready”; “Extremely Satisfied” to “Not At All Satisfied”). Open-ended questions allowed participants to provide further information on each aspect of the session in text boxes with no word or character limit.

To enhance therapist administration adherence of FC-FORT, the group facilitators were provided with a weekly 30-minute supervision with the study's lead psychologist. In addition, all sessions were recorded and reviewed by the research coordinator using an updated version of the fidelity checklist used to evaluate adherence during the previous FORT studies (38–40, 53). If adherence was less than 80% on any session, the research team provided additional feedback and supervision to the group facilitators.

Post-intervention, FC and therapists were invited to take part in a brief semi-structured exit interview (30–60 min) with the research coordinator. To maximize participation, FC were compensated 20$ for their interview time. Interviews were recorded via Zoom and then transcribed verbatim by the second author and/or using NVivo Transcription. The content of these interviews and questionnaires were analyzed, summarized, and presented back to the advisory board to further refine the FC-FORT content and format.

#### 2.4.4. Data analysis

Quantitative data were summarized using descriptive statistics. Qualitative data were analyzed using conventional content analysis (54). Once transcribed, transcripts were read repeatedly by the first author and the second author to obtain an overall sense of the data. Exact words/sentences were then highlighted to capture key concepts discussed by participants. Transcripts were systematically coded into anticipated (i.e., motivations to participate, benefits of participation, expectations) and emergent codes by the first author, in collaboration with the second author. This was an iterative process whereby an initial set of themes were coded, applied to new transcripts, and revised to adjust for new information, until no new codes emerge. These codes were then sorted into subcategories and then into a smaller number of categories by the first two authors, with disagreements being resolved through discussion.

## 3. Results

### 3.1. Round 1

In total, 6 female FC demonstrated interest in participating in the study, were interviewed, deemed eligible, and provided consent. Of those 6, 1 FC withdrew her consent before the start of the group and 1 FC was unable to participate at the time of the group but demonstrated interest in participating later on. Ultimately, 4 female FC (66% of initially recruited participants) and 3 therapists (2 facilitators and 1 back up) participated in the first round of FC-FORT (January 28, 2022, to March 11, 2022). The mean age of FC was 51 years old (SD = 11.67; range = 32–63) (Table 4). Most (*n* = 3) were taking care of partners, whereas 1 FC was taking care of a parent. Survivors were diagnosed with prostate (*n* = 2), melanoma (*n* = 1), or pancreatic (*n* = 1) cancer. Their mean score on the caregiver version of the FCRI-SF was 25.3 (SD = 4.4; range = 21–32). FC described fears related to: losing their loved one and the impact of their deaths on other family members, repercussions of additional cancer treatments on loved one's health and quality of life (i.e., pain), and increased caregiver burden. FC were mainly White (*n* = 3), in a marriage/common law relationship (*n* = 3), employed full-time (*n* = 3), and had higher education (*n* = 3). FC rated their loved one's perceived risk for cancer recurrence as equal to (*n* = 2), more likely (*n* = 1) or much more likely (*n* = 1) than other persons (Table 5). Motivations to participate included gaining information and skills to manage their FCR, giving back to research, and helping their loved one manage their FCR. Average FC participation rate was 86% (5 sessions had 100% attendance, 1 session had 75%, and 1 session had 50%). Average therapist administration fidelity rating was 84.5%. All sessions had a fidelity rating of 80% and above, except for session 4 (65% rating) due to the health care professional's visit taking longer than planned. Similarly, the primary issues with the administration fidelity were time related (i.e., therapists had to skip certain excercises). Average response rates for all (*n* = 7) session feedback questionnaires combined were 68% for FC and 71% for therapists. All participants took part in the exit interviews.

**Table 4:** Sample characteristics (*N* = 10).

Variable	Values
Age (years), mean (SD)	51.6 (10.46)
Ethnicity, *n* (%)	
White	9 (90%)
Asian	1 (10%)
Province, *n* (%)	
Ontario	5 (50%)
British Columbia	5 (50%)
Marital Status, *n* (%)	
Single/Never married	2 (20%)
Married/Common-law	8 (80%)
Level of Education, *n* (%)	
Part of university/college	2 (20%)
University/college	4 (40%)
Graduate school	3 (30%)
Occupational Status, *n* (%)	
Unemployed	1 (10%)
Unemployed due to illness	1 (10%)
Employed part-time by choice	1 (10%)
Employed part-time due to illness	1 (10%)
Employed full-time	3 (40%)
Student	1 (10%)
Retired	2 (20%)
Annual Income Level, *n* (%)	
$00,000–$20,000	1 (10%)
$41,000–60,000	2 (20%)
$61,000–80,000	2 (20%)
Greater than $100,000	4 (40%)
Cancer Type, *n* (%)	
Prostate	3 (30%)
Pancreatic	1 (10%)
Melanoma	1 (10%)
Acute myeloid leukemia	3 (30%)
Hodgkins Lymphoma	1 (10%)
Sarcoma	1 (10%)
Stage, *n* (%)	
Gleason score of 9	1 (10%)
Gleason score of 7	1 (10%)
III	1 (10%)
IIA	1 (10%)
Unknown	6 (60%)
Treatment Received, *n* (%)	
Surgery	3 (30%)
Chemotherapy	3 (30%)
Chemotherapy & radiation	1 (10%)
Surgery, chemotherapy	1 (10%)
Surgery, radiation, adjuvant chemotherapy	1 (10%)
Relationship with Patient/Survivors, *n* (%)	
Spouse/partner	7 (70%)
Parent	2 (20%)
Child	1 (10%)
Receiving other psychological support, *n* (%)	
Yes	2 (20%)
No	7 (70%)
Living with a chronic medical condition	
Yes	4 (40%)
No	5 (50%)

**Table 5:** Perceived risk of their loved one's recurrence.

Question:	Compared to persons of their age, how do you rate your loved one's perceived risk of cancer recurrence?				
	Much less likely	Less likely	Equal to	More likely	Much more likely
	0	0	4 (40%)	2 (20%)	3 (30%)

#### 3.1.1. Feedback questionnaires

Overall, FC and therapists agreed to strongly agreed that FC-FORT sessions were useful, usable, desirable, accessible, and valuable (See Tables 6, 7). They agreed that the FC-FORT virtual format and the activities at each session were helpful to better understand and manage their FCR. They also agreed that sessions included a satisfactory amount of information (i.e., felt neither over or underwhelming). FC and therapists reported being very satisfied and rated FC-FORT sessions as very ready for end users. Therapists also reported a need for this program (i.e., few interventions offered to FC experiencing FCR despite their distress). Overall, the most acceptable sessions were 1, 2, 6, and 7. The least acceptable were sessions 3, 4, and 5 as they were rated by participants with only moderate satisfaction due to lack of perceived value, accessibility, and readiness. Qualitative data from the feedback questionnaires identified that organization/flow of session (session 3), the healthcare professional's visit (presentation by a registered nurse, with longstanding experience in psychosocial oncology, about symptoms of recurrence in various cancer types; session 4) and the emotional difficulty of exercises and break out room (session 5) impacted their overall scores.

**Table 6:** Round 1 & 2—Family caregivers’ session feedback questionnaires (mean scores).

	Session 1		Session 2		Session 3		Session 4		Session 5		Session 6		Session 7		Overall	
	R1	R2	R1	R2	R1	R2	R1	R2	R1	R2	R1	R2	R1	R2	R1	R2
Useful	4.125	3.75	4.25	3.88	4.25	4.5	3.75	4.13	4	3.3	4	4.08	4.5	4.25	4.125	3.983
Usable	4.625	4.83	4.875	4.38	4.5	4.7	4	5	4.5	4	4	4.67	4.5	5	4.429	4.654
Desirable	4.25	4.17	4.75	4.25	4	4.2	3.5	4.75	4	3.8	4	4.33	4	4.5	4.071	4.286
Accessible	4.375	4.42	4.625	4	3.5	4.2	3.75	4.75	4	3.8	4	4.25	4.25	4.25	4.071	4.238
Features	4.917	4.08	4.75	4.25	3.833	4.07	3.833	4.17	3.833	3.53	4	4.17	4	4	4.167	4.040
Satisfactory amount of information and tools	2.875	2.834	2.875	2.75	2.5	2.7	2.5	2.5	2.75	2.2	3	2.833	3	3	2.786	2.668
Valuable	4.125	3.83	4.625	3.75	3.5	4.1	3.375	4.25	3.75	3.4	4	4.17	4.75	4.25	4.018	3.964
Readiness	4	3.67	4	3.75	3	3.8	3	3.75	3.5	2.6	4	4.17	4	4.5	3.643	3.748
Overall satisfaction	4.25	4.33	4.25	3.75	3	4.5	3.25	4.5	4	3	4	4.17	4	4	3.821	3.964
Addition/removal of content^a^	1.08	1.333	1.33	2.25	1.667	2	1.833	2	1	1.8	1	1.167	1.667	1.5	1.368	1.607

All scores are out of a 5 point Likert Scale (“Strongly Agree” to “Strongly Disagree”; “Extremely Ready” to “Not At All Ready”; “Extremely Satisfied” to “Not At All Satisfied”).

^a^ Scores are out of a 3 point Likert Scale (“No”, “Maybe”, “Yes”).

**Table 7:** Round 1 & 2—Therapists’ session feedback questionnaires (mean scores).

	Session 1		Session 2		Session 3		Session 4		Session 5		Session 6		Session 7		Overall	
	R1	R2	R1	R2	R1	R2	R1	R2	R1	R2	R1	R2	R1	R2	R1	R2
Useful	4.25	5	4.5	5	5	5	4.5	5	5	5	5	4	5	4.5	4.75	4.79
Usable	4.25	5	5	4.75	5	5	3	5	4	5	5	4	5	4.5	4.46	4.75
Desirable	4	5	5	5	5	5	3.5	5	4	5	5	4	4	4.5	4.357	4.79
Accessible	3.75	5	5	5	4.5	5	3	4.75	3.5	4.5	5	4	5	4.5	4.25	4.68
Features	4	4.5	4	4.5	5	4.83	3.667	4.83	4	3.5	4.333	3.67	3.333	4.5	4.048	4.33
Satisfactory amount of information and tools	2.5	3	3	3	3	3	3	3	3	3	3	3	3	3	2.929	3
Valuable	4.833	5	4	5	4.667	5	3.833	5	4.667	5	5	4	4.833	4.5	4.548	4.79
Readiness	3.5	5	4	5	5	4.5	2.5	4.5	4	5	5	4	3.5	4.5	3.929	4.64
Overall satisfaction	3.5	5	4	5	5	4.5	3.5	4.5	4	4	5	4	4.5	4.5	4.214	4.5
Addition/removal of content^a^	1.833	1	1.667	1.1675	1.333	1.5	1.667	1.167	1	1.33	1	1	1.333	1	1.405	1.167

All scores are out of a 5 point Likert Scale (“Strongly Agree” to “Strongly Disagree”; “Extremely Ready” to “Not At All Ready”; “Extremely Satisfied” to “Not At All Satisfied”).

^a^ Scores are out of a 3 point Likert Scale (“No”, “Maybe”, “Yes”).

#### 3.1.2. Exit interviews

Qualitative analysis identified 4 key results: participant's experience with the format, satisfaction and engagement with the content, group cohesion, and perceived impact of FC-FORT according to participants.

##### 3.1.2.1. Participant's experience with the format

Overall, FC and therapists expressed that the length of FC-FORT was appropriate. Whereas some FC indicated that they might have appreciated more sessions, they also agreed that seven weeks allowed for enough time to cover the content, get to know the group, and explore their FCR. No participants indicated that it should be shortened.

“*I think it was appropriate. I think there's room if you wanted to go up to 8 weeks, but at the same time, the way that the sessions are organized and laid out, because you follow the structure, you’re not really pushing things along in a subsequent week and I think that 7 sessions is appropriate. I think it*'s much better than if it was going to be 6 weeks. I think 6 weeks would be a bit challenging.” [Therapist 2 (T2)]

Both FC and therapists agreed that 90-minutes were not enough to comfortably cover the content of each session. In some cases, FC reported that they did not feel as though they had a chance to completely engage with an exercise. In other cases, some exercises were skipped completely.

“*We always ran out of time and I don't think anyone was sitting there “I wish this would end”, we were all engaged from the beginning to the end, but again, that might’ve been sufficient if we didn't go through the homework or each checking in as to whether or not we went through the homework but there were some things in the booklet that we didn't go through because we didn't have time and it wasn*'t picked up in the next session” (P2).

In terms of timing, FC indicated that Friday sessions were convenient as it provided a chance to debrief over the weekend. However, having sessions during a workday created some barriers for FC who worked fulltime.

*“If it was in the evening yeah, no worries. But I work full time, so […] I’m only available in the evenings and weekends so it was a struggle for me to do every session because I only have a break for an hour and the sessions were 1:30 so I had to extend my day or take time off to do the sessions.”* (P2)

Finally, FC and therapists agreed that the virtual format increased the accessibility to FC-FORT as it was practical and convenient. In addition, a few FC mentioned that having the group virtually provided an additional sense of safety which increased their emotional vulnerability and allowed them to further explore and challenge their FCR.

*“It was actually good, doing it online it gives me a sense of distance and safety. I am present and I can see people […] That's really important when you’re sharing anything that personal. Being online, I’m in the safety of my own home, I can step away at the end of it and I don't have to drive home if I’m emotionally rot, I didn't have to set aside an hour to get downtown to get to the meeting and then an hour after.”* (P5)

##### 3.1.2.2. Satisfaction and engagement with the content

FC and therapists reported feeling very satisfied with FC-FORT (i.e., workbook, content, coping strategies). Preferences varied in terms of which exercises FC found most helpful; however, relaxation/mindfulness exercises (e.g., mindful eating, guided imagery) seemed to have been preferred to cognitive ones (e.g., thought log). Some FC mentioned that it was the combination of all the tools that promoted change.

*“There were certainly some exercises in there that were useful but none of them on their own would have given me the “ha-ha” moments that I’ve had.”* (P5)

Of note, while session 5 was the least highly related according to the feedback questionnaires, all FC mentioned that it stood out as being the most valuable to address their FCR as it allowed them to concretely identify/name their fears (often for the first time), rate them in terms of intensity, and openly share and discuss them with the rest of the group and facilitators. This suggests this session, while helpful, was particularly challenging for FC.

*“There was one [session] where we were kind of rating our fears and concerns and after I did that, I looked at it and I didn't realize that [those fears were] going on. It really jumped out at me when I was dealing with it because I hadn't really thought about it in an organized way.”* (P5)

FC also noted that group support mechanisms (e.g., conversations and exchanges) had significantly contributed to a better understanding of their FCR, normalized their caregiving experiences, and increased their sense of coping (i.e., validating their difficulties, learned new coping strategies).

*“I think even listening to the other women and their fears and coping techniques and what they do to recharge their battery, it brings the tone that we’re worthy and ‘oh I never thought of doing that to recharge my battery, that's quicker’ and ‘oh I think I’ll try that’. Kind of made an impact.”* (P6)

FC and therapists did identify some issues with the FC-FORT content. Primarily, the health care professional's visit (session 4) was deemed unhelpful by almost all participants due to diversity in types of cancer and participant's locations (i.e., each province has its separate guidelines and procedures), and the information provided (i.e., too generic).

*“You have people from across the country […] So, unless you have a representative for each of the provinces of the people who are participating in the group who can speak to the local services, it's not really helpful. Because the information about the actual cancers, there's a multitude of information from the cancer agencies, Cancer Canada, everything, I hadn't come across the symptoms of when it reoccurs because most of it talks about the cancer when it's happening, but the actual having a healthcare provider, I didn't really find that valuable.”* (P2)

In addition, the organization of certain sessions and the use of the breakout room were seen as reducing the usability of FC-FORT and participants' overall experience. For example, in some sessions (e.g., session 3), some participants found that the exercises did not flow well together and led to confusion. P6 described this session as “very up and down”. FC also expressed that they would have preferred to remain as a group for session 5 and that they found the breakout room to be out of place or isolating.

*“There's one thing that I definitely think needs to be changed. When we went through our worst feelings or the worst fears that we had we were given breakout rooms to write that down and the breakout rooms were alone and […] I think that that particular exercise is too emotionally intense to leave people on their own.”* (P2)

Finally, FC experiences with the homework and logs (both for the cognitive and mindfulness exercises) were mixed. While some participants appreciated the logs as it gave them an opportunity to reflect upon and articulate their feelings, some found that they were not helpful for the management of their FCR or were not easy to complete (i.e., confusing, language use was not patient friendly). In addition, most participants reported that they often forgot or were too busy to complete the homework throughout the week.

*“[…] The thought log was not for me. I think part of that was the way it was written. The “how I felt before 0%–100%, how I felt after” it kind of didn't make sense to me. […] it was more frustrating than anything so that's why I gave it up pretty much right away.”* (P5)

##### 3.1.2.3. Group cohesion

Overall, FC and therapists indicated that they experienced trust, acceptance, sharing, understanding, connection within the group. For example, P6 stated: *“I really didn't think it would click quite the way that it did being online and not in person. I didn't quite think that it would be unity feeling.”*. Furthermore, T2 felt that *“the cohesion was there, I think they were genuinely happy to see one another and to hear one another's update and they cared what everybody else was doing, they were okay to share so, when I think of the mark of did, we reach to kind of measure cohesion.”*.

In addition, FC described feeling supported, validated, and included by both therapists throughout FC-FORT. Most FC also indicated that the therapists' skills (i.e., time and group management, knowledge) facilitated overall group cohesion. On their end, therapists reported feeling that the weekly check-ins contributed to enhancing group cohesion.

*“I think [the therapists] worked well together […] and they both were so good at making you feel comfortable about whether you were snot crying […] or having a moment or the epiphany moment, they were so good at working through all of that with you, both of them. It was great.” (P6)*

While FC indicated that they appreciated the smaller group size, as it allowed for deeper personal connections and made it more comfortable to share personal information, some FC noted that the somewhat limited diversity within the group (i.e., caregiving role & experience, age, cancer types, coping strategies) occasionally made it difficult for them to connect with others.

*“I was pleasantly surprised by the size of the group; I thought it was going to be a lot more people and that would’ve been more difficult for me. In some sense it would’ve been more difficult, in others it would’ve felt less difficult in that there's a bit of opportunity to not have to share and interact. I would always do that, I think also having more individuals, you get a broader perspective. […]. I was going to say, because it's such a small group, there wasn't a whole lot of experiences that matched mine, they were sort of on one side and I was on the other.”* (P2)

Finally, all participants indicated that the virtual format of the group did not affect their sense of connection or group cohesion.

*“I was honestly surprised, I was expecting to feel a little less of a group and more single people doing this but just doing it at the same time rather than a group, but it worked, we became that group and it worked as a group. I can't say that being online made it any different.”* (P6)

*“I didn't feel like I lost any of the personal connection. I think it's a brilliant way to do any groups in the future.”* (P5)

##### 3.1.2.4. Perceived impact of FC-FORT on participants

All FC reported that FC-FORT has had a positive impact on their lives. Most noted that they now had a better understanding of their FCR, that they had gained tools (i.e., understand and recognize triggers, recognize their biggest fears, relaxation, and mindfulness exercises) to better manage their FCR and that FC-FORT had had an impact on their stress/anxiety (i.e., felt calmer about their fears) personal relationships, the stress they experience at work, and their quality of life.

*“I feel like I’m much better able to understand why that fear is so hard for me and what's really at the root of it and once I know what my triggers are then I know how to deal with them so that was the most important gain from this group.”* (P2)

When asked how important the group had been for them [(on a continuum from 0 (Not important) to 10 (Extremely important)], FC rated, on average, the importance of FC-FORT in their lives as 9. In addition, all FC indicated that they would recommend FC-FORT to others.

*“I would highly recommend this to somebody coming into it because right away let's get the coping techniques for you into practice and get you that and make sure that you’re in a good spot.”* (P6)

Finally, FC indicated that they planned to continue using coping strategies learned through FC-FORT or review the workbook as needed. FC identified consistency as their primary obstacle to implementing these changes long term.

*“There's a lot of reflective questions that are in the workbook and I try to take the weekends to focus in more on this type of reflective work and it helps to ground and center me.”* (P4)

*“I know that there will be a challenge when it's going well not to do the relaxation and the mindfulness and then, all of a sudden, you’re in a panic situation and then you have to come back to it and relearn it and re-practice it.”* (P6)

#### 3.1.3. Adaptations and changes

Results from this initial usability round were presented back to our advisory board in April 2022. Based on results and expert feedback, a second round was deemed necessary. To address participants primary concerns, key adaptations were made to FC-FORT including increasing length of sessions from 90 to 120 min, removing and reorganizing exercises (e.g., removing the health care professional's visit, changing order of exercises within sessions), modifying some of the virtual features (e.g., instead of separating participants into individual break out rooms that therapists then entered, all participants stayed in the main Zoom as a group and an individual break out room was created for a therapist. FC were sent one at a time to see the therapist, while the rest of the group stay in the main Zoom), and changing some of the workbook's written content (e.g., softening language, adding examples, modifying questions to increase standalone comprehension). Please see Table 2 to see an exhaustive list of suggestions and modifications.

### 3.2. Round 2

In total, 8 female FC demonstrated interest in participating in the study, were interviewed, deemed eligible and 6 provided their consent. Of the initial 8, 1 FC declined to participate due to scheduling conflicts and 1 FC was unable to participate at the time of the group but demonstrated interest in participating later on. Ultimately, 6 female FC (75% of initially recruited participants) and the same 3 therapists (2 facilitators and 1 back up) from round 1 participated in the second round of FC-FORT from May 19, 2022, to June 29, 2022. The mean age of FC was 52 years old (SD = 10.46; range = 21–68) (Table 4). Most (*n* = 4) were taking care of partners, whereas 2 FC was taking care of adult children. Patient cancer types included leukemia (*n* = 3), lymphoma (*n* = 1), prostate (*n* = 1) or sarcoma (*n* = 1). FC were mainly White (*n* = 5), in a marriage/common law relationship (*n* = 5) and had higher education (*n* = 5). Their mean score on the caregiver version of the FCRI-SF was 24 (SD = 3.48; range = 21–29.5). FC described fears related to: losing their loved one, repercussions of additional cancer treatments on loved one's health, quality of life (i.e., pain) and future, and increased caregiver burden and impact on FC future plans. FC rated their loved one's perceived risk for cancer recurrence as equal to (*n* = 2), more likely (*n* = 1) or much more likely (*n* = 2) than other persons (Table 5). Motivations to participate included gaining information and skills to manage their FCR and giving back to research. Average FC participation rate was 81% (4 sessions had 100% attendance, 2 sessions had 66.7%, and 1 session had 33.3%). Therapist average administration fidelity rating was 89%. All sessions had a fidelity rating of 80% and above. Average response rates for all (*n* = 7) session feedback questionnaires combined were 76% for FC and 85.7% for therapists. All participants took part in the exit interviews.

#### 3.2.1. Feedback questionnaires

Results obtained from this second round resembled those of the first (See Tables 6, 7). Overall, FC and therapists reported being very satisfied with FC-FORT. FC rated FC-FORT sessions as very ready for end users, while therapists rated them as extremely ready. Of note, session 5 received the lowest rating from FC (Table 6) with readiness and satisfaction being rated as moderate. In addition, FC indicated “Neither agreeing nor disagreeing” with the value of session 5. Qualitative data from the feedback questionnaires identified that the readiness of participants to face these fears and the difficulty of the exercises themselves impacted the overall rating.

#### 3.2.2. Exit interviews

Similar results as Round 1 emerged: participant's experience with the format, satisfaction and engagement with the content, group cohesion, and impact of FC-FORT on participants. However, only themes relevant to the modifications made after Round 1 will be highlighted below.

##### 3.2.2.1. Participant's experience with the format

FC and therapists stated that they appreciated the format of FC-FORT. FC and therapists still expressed that the overall length of FC-FORT was appropriate and agreed that 120-minutes was enough to comfortably cover the content of each session and have in-depth discussions. In addition, this allowed for a break midway through the sessions—which was appreciated by FC.

*“First, I thought it was going to be pretty long […] but the time flew by. […] it was never like, ‘Oh, when is this going to be over?’ It was like, ‘Oh boy, are we ready for a break already?’ […] And it was really good having the bathroom breaks partway through.”* (P7)

FC and therapists agreed that the virtual format increased their accessibility to FC-FORT in terms of practicality, flexibility, and convenience. In addition, most FC expressed having appreciated the opportunity to connect with other FC across Canada.

*“[…] For me, the online format works absolutely beautifully. What I also like is being able to have opinions from people in different provinces […] having that cross-Canada [perspective] is kind of interesting as well so I love the online format.”* (P10)

##### 3.2.2.2. Satisfaction and engagement with content

FC and therapists reported feeling very satisfied with FC-FORT (i.e., workbook, content, coping strategies). Once again, preferences varied in terms of which exercises FC found most helpful, with relaxation/mindfulness exercises being slightly more preferred than cognitive ones. Of note, most FC mentioned that session 5 (i.e., writing down worst fear) stood out as being the most valuable to address their FCR.

*“Writing out that worst case scenario really stands out.[…] I was actually able to get it down on paper so that I could read over what was floating around in my brain, in my thoughts […] as if I was just an observer […]. Somehow seeing it written down […] it helped me to pre deal with emotions and to see a different perspective of it.”* (P8)

However, some FC had trouble engaging in session 5, specifically the writing of the worst-case scenario, due to the challenging nature of the exercise and their individual situations.

*“I found this session very upsetting actually. I know it was meant to be in many ways, however I was not emotionally ready to take part in some of the exercises.”* (P11)

*“The tools and conversation were useful but the writing out our biggest fears was hard and I'm not sure if it would be a great exercise for everyone.”* (P4)

All FC indicated that group conversations had contributed to normalizing their fears and increasing their sense of coping

*“I gained so much comfort from having a space where people talked about this freely because really in my day-to-day life, everyone around me is aware of what's going on, but I would never just like openly or comfortably talk about […] how scary it is and expect them to understand. Versus this space, everyone really did understand and was dealing with it in their own way. […] But it was it was nice to get people's advice and have people properly understand you.”* (P4)

*“I think just the solidarity of hearing other people's experiences was great because I've been very kind of isolated […].”* (P10)

Consistently completing homework continued to be the primary challenge for FC. However, most stated that the weekly homework debriefs provided another chance to engage in the homework as it led to group discussions regarding various topics and learn about others' experiences—regardless of if they had completed the homework themselves. FC indicated that being able to refer to the homework assignments in the future or at a more convenient time was an additional bonus.

*“The fact that they were available and then that the discussion that followed was never dependent on whether the group did [the homework; …]. Going over, say what the homework was, because I can think of at least once where I hadn't even looked at it and it didn't matter because it was brought into the conversation in such a with such good explanation. And then the conversation, the session just flowed from there. So, it's there, I think it's really good that it's there in the workbook, but you know, doing it after the fact isn't going to take away from its effectiveness.”* (P8)

Finally, two FC expressed that the use of the one-on-one breakout room with one of the group therapists negatively affected their overall experience in Session 5. In fact, FC mentioned that the short time allotted to each participant made them feel rushed and unheard.

*“I found the breakout sessions confusing and a bit abrupt. Not sure if I fully understood what they were for. […] Either more clarity on use of breakout sessions, or more time.” (P8)*

*“[…] The only session that I came out feeling not so good was when there was one session where we broke out into individual groups like one on one if we wanted to talk to [the therapists]. But it was so timed and short lived that it actually triggered me about being cut off, about wanting to talk about it. And I think if you're going to go one on one, you have to be able to allow a little more time.”* (P9)

##### 3.2.2.3. Group cohesion

Overall, FC and therapists indicated that they experienced good group cohesion. In fact, P8 expressed that they appreciated the “*level of connectedness*” that was established as early as the first session. In addition, T2 expressed “*when I think back to this specific group, I actually think we had a really […] cohesive group, a very rich group, there was the support for one another that was really there”*. FC indicated that participant's openness and vulnerability contributed to the group cohesion.

*“I felt like people connected. I felt […] there was meaningful conversation. I think it definitely exceeded what I expected in those ways. It felt like everybody participated, no one monopolized all the conversation or some of the things you get sometimes in groups.”* (P11)

Both FC and therapists found the group size allowed participants opportune time for expression and supported the creation of a safe and mutually supportive environment to engage in each exercise according to their needs/comfort levels and increased diversity within the group.

*“This group of ladies from across Canada with different cancers, different parts of the cancer journey and different cancers period and different stages of life that we’re all at. And I thought, who’d have thought that there'd be this group and it didn’*t take long to really feel *‘*I’m not the only one with these thoughts’, and that was actually it was really comforting.” (P8)

*“If some people don’t come, I feel like the size of the group is still good. And also sometimes I didn’t want to talk if there's a question that I just didn’t resonate with and […] so a six people like you kind of have the freedom of doing that […].”* (P4)

Finally, participants indicated that, overall, the virtual format of FC-FORT did not affect their sense of connection or group cohesion (i.e., allowed for exchanges, unstructured conversations, shared experiences). FC indicated that therapists' time management abilities, capacity to include all members in conversation, and weekly check-ins contributed to the success of the virtual platform.

*“I found [the therapists] directed it really well and made sure everybody got a chance to speak. Even if they asked them if they wanted to share anything and they didn’t want to, they were okay with that too, but everybody usually said something when given the opportunity.”* (P9)

*“Professionally guided discussions and safe environment for us all to share and it really created […] a very cohesive group.”* (P8)

Challenges with cohesion were primarily related to participant attendance (i.e., missing sessions or joining from different settings) and participation during the group (i.e., natural tendencies to talk more or less than others, willingness to engage in exercises).

*“I don't know of anything that hindered it. It's hard if people missed some weeks, but that can't always be helped. I felt more connected to the ones that were there more often […].” (P7)*

*“I do think attendance was difficult. And I do think having sometimes folks either just be on the phone and not be on video or being in the car. I think those things kind of stopped group cohesion. I think it's still happened. I think it's still existed.”* (T1)

## 4. Discussion

This study aimed to (1) adapt the FORT intervention for FC living with FCR (FC-FORT) and to a virtual format and (2) test its usability. A multidisciplinary advisory board composed of the research team, community therapists and FC was recruited and oversaw the adaptation of FORT and its patient and therapist manuals. Based on their expertise and feedback, many minor and major adaptations were made. Notably, additional exercises aimed at addressing FC's self-care, overcoming protective buffering by having difficult conversations with loved ones regarding FCR, and discussing and optimizing the use of their loved ones' health care teams were added. As a result, a seventh session was included to accommodate this added content. FC-FORT remains, like FORT, a standardized and manualized therapist-led group intervention aimed at addressing FCR in FC through mindfulness exercises, cognitive-existential therapy, and principles of group therapy. However, a major recommendation was for FC-FORT to be offered as a synchronous, online, virtual group via the platform Zoom, instead of a face-to-face group.

Following these adaptations, two rounds of usability testing ensued. Based on participant feedback, further modifications were made to FC-FORT. Namely, the session length was increased from 90 to 120 min, exercises were removed or reorganized, and some of the patient manual's written content and virtual features were modified. Overall, results from both rounds demonstrated that FC and therapists found FC-FORT to be useful, usable, desirable, accessible, and valuable. In addition, they reported high levels of satisfaction with both the content and the virtual format of FC-FORT, felt that FC-FORT was ready for use and indicated that they would recommend it to others. Given these results, FC-FORT is deemed ready for pilot testing (See Table 8 for an overview of FC-FORT). A key contributor to the successful adaptation of FORT to FC-FORT is the contribution of valuable FC insight in both the adaptation and usability testing of the intervention. In fact, including FC in research could be considered a best practice as it leads to meaningful additions to the scientific literature as well as improved health outcomes (45). However, a 2019 systematic review (55) found that a large number of studies dedicated to cancer FC interventions did not in fact include FC input in the development and evaluation of their interventions. It would seem that this study therefore distinguishes itself from previous interventions studies dedicated to FC.

**Table 8:** Overview of FC-FORT sessions.

Session 1: Introduction to the group, learning new skills to deal with FCR (120 min)	• Introduction by each participant with a focus on their experience with fear of cancer recurrence (FCR). • Introduce ABC model of therapy, FCR model, cognitive restructuring and identify FCR triggers. • Psychoeducation on the importance of self-care, ways to engage in self-care and potential obstacles. • Teach cognitive restructuring to challenge FCR related thoughts. • **Homework:** Complete thought log and practice self-care.
Session 2: Identifying knowledge gaps on FCR (120 min)	• Discuss uncertainty in FCR and caring for a loved one with cancer. • Discussing FC's knowledge regarding FCR, identifying gaps in knowledge and navigating the patient's health care team to obtain knowledge on FCR. • Teach progressive muscle relaxation (PMR). • **Homework:** Complete thought log and practice daily PMR.
Session 3: Increasing tolerance for uncertainty (120 min)	• Discuss acceptable level of worry. • Challenge faulty beliefs about benefits of worry (in relation to FCR). • Discuss uncertainty and ways of regaining a sense of control. • Teach the use of calming self-talk & introduce FC to relaxation files. • **Homework**: Challenge faulty beliefs about benefits of worry. Practice calming self-talk & PMR daily.
Session 4: Building your coping skills (120 min)	• Provide psychoeducation about worry and the need for exposure to worse fears. • Discuss maladaptive coping strategies used when experiencing FCR (focusing on avoidance). Group discussion and sharing of different coping strategies to address FCR. • Address communication difficulties with loved ones relating to FCR • Teach guided imagery. • **Homework**: Have a conversation about FCR. Practice guided imagery daily; challenge faulty beliefs about benefits of worry; complete thought record with behaviour.
Session 5: Getting deeper into underlying fears (120 min)	• Pink elephant exercise (i.e., demonstration on the impact of avoidance). • Promote emotion expression and confront specific fears that underlie FCR by writing down worse fear scenario. • Teach body scan exercise. • **Homework:** Read worst case scenario daily. Practice self-care & body scan exercise daily.
Session 6: Moving beyond specific fears (120 min)	• Review exposure to worst case scenario exercise. • Discuss ways of coping with some of the feared outcomes. • Encourage participants to become re-engaged with important life goals, people or activities despite FCR. • Discuss what meaning the future and planning now have for them. • Teach mindful eating. • **Homework:** Write down goals and priorities for the future, practice mindfulness daily..
Session 7: Review and conclusion (120 min)	• Review all content covered. • Discuss future goals and setting new priorities. • Promote the expression of saying good-bye to the group and provide closure.

One of the most notable findings from this study is FC and therapists’ appreciation for the virtual aspect of FC-FORT's adaptation. Overall, participants mentioned that the virtual format increased their accessibility as it was practical, flexible, and convenient. Some even mentioned that the virtual format provided them with an increased sense of safety. In addition, they expressed still being able to experience trust, acceptance, sharing, understanding, and connection with other group members (i.e., group cohesion) and therapists (i.e., therapeutic alliance). Alternatively, the primary issue reported by FC related to the virtual format was the use of the breakout room feature in Session 5. Altogether, our results suggest that while the fundamental components of group interventions are present when using a virtual format (i.e., group cohesion, therapeutic alliance, developing coping strategies to manage FCR), it seems that the “technical” aspects of the interventions (e.g.,: breakout rooms, participants keeping their cameras closed) may be less appreciated by participants/more difficult to adapt to a virtual setting (e.g.,: one on one time between therapists and participants). Therefore, while the virtual format provides many advantages for FC and was generally well received by participants, further reflection is needed on how to better adapt some specific aspects of face-to-face interventions to virtual settings.

Moreover, FC recruitment and attendance proved to be challenging. While our initial recruitment efforts were limited to the provinces of Ontario and Quebec, these yielded limited interest from the caregiver community. Given the well-documented difficulties (56) in recruiting this specific community (i.e., high levels of burden or accessibility issues) as well as limited capacity to recruit in traditional hospital settings due to the COVID-19 pandemic (i.e., caregivers no longer allowed in the hospital with patients) our team decided to expand our recruitment efforts across Canada. The virtual format of FC-FORT combined with the pandemic context (i.e., lockdowns, no accessible in person services, remote work) provided an ideal scenario for this change in recruitment and an opportunity to increase the intervention's accessibility. Interestingly, almost all FC expressed that had FC-FORT not been virtual they would not have been able to participate or would have been limited in their full participation. Moreover, many also expressed having appreciated the opportunity to connect with other FC from across Canada with whom they most likely would not have connected with otherwise. These results echo the previously established literature demonstrating that e-health interventions may effectively be more acceptable and feasible for FC (36, 37, 57). Additionally, the lessons learned throughout this study and the adaptations made to our recruitment strategy will also allow us to modify our recruitment efforts for the next phase of the project (i.e., the pilot study). Namely, we will continue to expand our recruitment nationally and will aim to further recruit through social medias and community support partners across Canada given that this is how most of our FC were recruited.

Finally, some studies (58, 59) have previously focused on the adaptation process of in-person interventions for FC to a virtual format. For example, Zulman and colleagues adapted an in-person intervention, aimed at improving communication between partners, to a virtual format. Similarly to the current study, their results demonstrated that many elements of in-person interventions can be successfully adapted to a virtual format and perceived as acceptable by dyads (patients and partners). A multidisciplinary team, including FC, was also used in the adaptation process further demonstrating the necessity of FC insight in research. Additionally, Northouse & colleagues evaluated the feasibility of adapting a previously in-person intervention for dyads and found that it was feasible (i.e., good retention rates, accessibility, and satisfaction) to offer this intervention using a virtual delivery format. However, less is known about the adaptation of validated interventions previously dedicated to cancer survivors to FC. Therefore, the results of this adaption of FORT to FC-FORT demonstrate the pertinence of adapting previously validated interventions dedicated to cancer survivors with specific FC related content. Overall, participants indicated that they appreciated the content of the manuals and found them to be pertinent to them/their experience. However, results from this study suggest that while FC reacted well overall to the intervention exercises (with some preferences for the mindfulness/relaxation exercises), what FC most appreciated from FC-FORT was the ability to go through the intervention material in a group format. More specifically, according to participants, FC-FORT provided an opportunity to speak openly about FCR with other FC and a chance to connect with others who are experiencing similar difficulties (in both FCR and caregiving). Noteworthily, while specific content was added to better reflect FC common difficulties, the elements of FC-FORT that were both most and least appreciated resembled what was reported in the original FORT validation study (60). For example, in the original FORT study, Session 5 was also reported as the most difficult session by cancer survivors but generally deemed the most useful. Moreover, unstructured conversations and exchanges amongst participants were also some of the most appreciated aspects of FORT. Similarly to FORT, primary challenges throughout both rounds included homework completion. This project is a first step in establishing the pertinence of adapting pre-existing FCR interventions dedicated to patients for FC with few modifications made.

## 5. Study limitations

Limitations of the current study include small group size (4 instead of intended 6–8) for the first round of usability testing. Moreover, not all FC used FC-FORT exactly as intended with most missing one or two sessions. In addition, as is common in psycho-oncology research with FC, most participants were White women taking care of male partners. Canadian representation was also limited with participants only coming from either British Columbia or Ontario. Future adaptations of this study should include diverse populations and formats (i.e., combined women and men, in person, hybrid). Given the current adaptation of FORT to a new population (i.e., caregivers) and to a virtual format, our team chose to limit the amount of adaptations in order to more accurately represent the usability, and eventual feasibility and acceptability, of FC-FORT. However, FORT is currently being adapted to Mexican breast cancer survivors (61). Similar adaptations of FC-FORT could be made to represent for men FC and other cultural adaptations. Based on the lessons our team learned throughout the study, future adaptations should include an advisory board of caregivers, researchers and health care professionals in order to appropriately adapt FC-FORT. This study, as well as upcoming pilot study, are the first steps in developing further adapations for diverse populations.

## 6. Conclusions

This study demonstrated the usability of the adapted FC-FORT with family caregivers and in a virtual format. Themes from the usability testing emphasized the importance of including FC in research and developing/adapting targeted and accessible therapist-led group interventions for Canadian FC living with FCR. Results indicate that FC-FORT's feasibility and acceptability is ready to be pilot tested in a mixed-method randomize-control trial.

## Data Availability

The datasets presented in this article are not readily available because of its proprietary nature. Requests to access the datasets should be directed to jlama023@uottawa.ca.
